# Effects of thymoquinone and the curcumin analog EF-24 on the activity of the enzyme paraoxonase-1 in human glioblastoma cells U87MG

**DOI:** 10.1080/14756366.2024.2339901

**Published:** 2024-06-12

**Authors:** Ender Simsek, Asuman Sunguroglu, Ahmet Kilic, Nurbanu Özgültekin, O. Ozensoy Guler

**Affiliations:** a Department of Medical Biology, Ankara Yildirim Beyazit University, Ankara, Turkey; b Department of Medical Biology, Ankara University Ankara, Turkey; c Multiscale Thermofluids School of Engineering, The University of Edinburg Edinburg, UK

**Keywords:** thymoquinone, curcumin analog EF-24, glioblastoma, PON1

## Abstract

The spices and aromatic herbs were used not only in cooking to add flavour and smell to dishes but also for medicinal use. Nigella sativa, also called black cumin, is one of the species that contains an important bioactive component, thymoquinone (TQ), which has antioxidant, anti-inflammatory, antimicrobial, and antidiabetic effects. Curcuma longa, which also includes curcumin, has numerous anti-cancer properties. However, the bioavailability of curcumin is lower than that of its analogs. An analog of curcumin (EF-24), which has better bioavailability than curcumin, is capable of exerting a high anti-cancer effect. In our study, we determined the effects of PON1 enzyme activity on the proliferation and aggressiveness of glioblastoma cancer treated with TQ and EF-24 from lysates of the glioblastoma cell line U87MG. The results were determined as increased PON1 activity after treatment with TQ and EF-24 in the U87MG cell line (*p* < 0.0001).

## Introduction

### Glioblastoma and PON1

Glioblastoma is one of the best-known brain tumours in adults^1^. Oxidative stress is attached with the pathogenesis of gliomas since oxidative stress produces reactive oxygen species (ROS) which are resulting to stimulate the cancer progression^2^. The enzyme paraoxonase (PON1; EC 3.1.1.2) hydrolyses paraoxon to eliminate its harmful effects^3^. The calcium-dependent metalloenzyme PON1, which has an antioxidant effect, is responsible for the HDL particle-mediated reduction in lipid oxidation of low-density lipoproteins. Paraoxonase is able to degrade paraoxon, chloropyrophos-oxon, and many other organophosphates in the blood plasma of mammals ^4^. The enzyme family of paraoxonases comprises 3 isozymes of paraoxonases 1, 2 and 3 (PON1, PON2, PON3); in humans, their genes are located in the 7^th^ chromosome (7q21.1). The three-dimensional structure of the paraoxanase with a molecular weight of 45 kDa is stabilised by a disulphide bond^5^. The six histidine residues and calcium ions are located in the active centre of the enzyme structure^6^. PON2 is widely distributed in all tissues. As an intracellular membrane-bound protein with a glycosylated tail, PON2 is also known for its relationship to cancer, alongside PON1 and PON3^7^. Paraoxonases are associated with cardiovascular disease, as the absence of the PON1 gene promotes atherosclerosis^8^. When PON1 is present in HDL, it protects low-density lipoproteins (LDL) from oxidative stress. The presence of the PON2 gene also has a similar antiatherogenic ability to prevent heart attacks ^9^^,^^10^. Brain tumours are classified into different types depending on the type of tumor^11^. For example, the types of gliomas and meningiomas are localised differently in men and women, respectively^12^.

The reasons for brain tumours are related to some environmental claims that agriculture is responsible for the mechanisms of tumorigenesis ^13^. High PON1 activity is indicated by various polymorphisms such as dietary and lifestyle factors and also environmental chemicals. PON1 is associated with high-density lipoprotein (HDL) and this ability of PON1 plays a crucial role in lipid metabolism^14^^,^^15^. This role results in PON1 preventing the lipid-soluble radicals of lipid peroxidation.

The cerebral microvessels serve as a barrier to protect the brain from chemical influences^16^. Unfortunately, so many effectors, such as drugs, organophosphorus insecticides, and other xenobiotics, can penetrate the brain barrier. Thanks to this barrier system, cerebral microvessels are also able to metabolise xenobiotics to avoid reactive oxygen species^17^. If we think about the state of PON1, it should act at this level as a detoxifying enzyme to hydrolyse activated intermediates of some organophosphorus compounds^18^^,^^19^. Consequently, the PON family of enzymes has important capabilities in stimulating low oxidative stress levels associated with inflammation and cell death in cancer patients. The regulation of the redox system by PON1 in tumour cells suggests that PON1 has a close relationship between inflammation and oxidative stress in different types of carcinomas^20^.

### The importance of herbs in cooking

In the cuisines of the world, some countries have a variety of cuisines that use different herbs to change the taste or colour of the food. Turkish cuisine is also one of the major cuisines in which herbs are used for seasoning. The use of herbal plants as medicine in Anatolia or in other ancient regions is historically conditioned. This is because the diverse uses of medicinal herbs are still an attractive area today^21^. The aromatic herbs are used as spices in food or for other purposes ^22^. If we look for the path of spices in the world, we can easily understand the importance of the spice route throughout history.

*Thymus vulgaris,* the thyme plant, belongs to the Lamiaceae family and is also a popular herb because of its incredible smell. The thyme plant is known for its antimicrobial, digestive, antispasmodic, and antioxidant properties^23^^,^^24^. One study indicated that thymoquinone, TQ, is attractive by targeting the PPAR-γ signalling pathway to prevent cancer cell invasion in breast cancer ^25^. The possible role of TQ in the demethylation of cancer cells shows that TQ increases PTEN expression when inhibition of phosphorylated Akt occurs^26^. A curcumin analog of EF24 is involved in the apoptosis process^27^. This activation is also maintained by reactive oxygen species (ROS)^28^. EF24 thus has several crucial roles in influencing the signalling pathways of NF-ҡB, HIF-1α, p53, STAT3, miRNAs, and ROS mediators^29^.

### Cancer and medical herbs

A scientific article has nothing to do with the findings that herbal plants have a preventive effect against cancer. However, there are studies that indicate the anti-cancer effect of some plants compared to conventional chemotherapeutic agents^30^. When it comes to the side effects of drugs, there are always a number of possible comparisons with herbal medicines. Certainly, herbal medicines have a lower risk of side effects than standard medicines because they are not produced by chemical reactions, as is the case with targeted therapy, for example. They can be used for mild illnesses in the same way as plant extracts. There are some studies that suggest a bridge is maintained between nutrients and metabolic pathways that are focused on health^31^. Spices, which have low toxicity, are a useful tool for an appropriate diet that makes it possible to reduce the risk of some diseases.

### ROS and redox relationship with PON1 enzyme

Oxidative stress and its definitive product, free radicals, and ROS species are the closest neighbours of tumours. This neighbourhood can cause a cancer relationship and lead to disease progression. However, the amount of ROS promotes DNA, protein, and lipid damage, genetic instability, and tumorigenesis^32^^,^^33^. To break this correlation and prevent tumorigenesis, antioxidant levels can be linked to the antioxidant enzyme PON1. Most of the researchers postulated that PON1 acts as a scavenger enzyme in the formation of oxidised low-density lipoprotein (LDL) and, in this role, prevents the formation of oxidised phospholipids. This ability interrupts the oxidation of phospholipids into HDL by the PON1 enzyme^4–6^. This function of the antioxidant enzyme PON1 not only leads to the prevention of fat-soluble radicals, but also to the reduction of cancer development^17^^,^^18^.

## Materials and methods

### Materials

AZA (acetazolamide), TQ, and EF 24 were purchased from Sigma-Aldrich (St. Louis, MO, USA). The glioblastoma cell line U87MG was purchased from the American Type Cell Collection (ATCC, Washington, DC). Each substance has a purity of ≥99%

### Methods

#### Cell culture

0.25% trypsin–EDTA was used to recover U-87 MG glioblastoma cells suspended in Roswell Park Memorial Institute 1640 medium containing 15% FBS, 1% L-glutamine, and 1% penicillin–streptomycin. The cells were placed in 25 cm^2^ flasks. They were then transferred to 96-well plates. Each well contained 100 ml of medium with 1 × 105 cells. As a control, only 150 μl Eagle medium (Gibco Sigma) were added to each, and then the MTT assay was performed^34^.

#### PON1 enzyme activity

The PON1 enzyme activity is determined spectrophotometrically at 412 nm ^35^. A 100 mM Tris–HCl with 2 mM CaCl_2_ and a 2 mM paraoxon solution were each prepared as a buffer for the basic activity and as a substrate solution. In the next step, the cuvette is fixed with the basal activity buffer, the substrate solution, and the cell lysates. The absorbance values of the test samples were determined at 37 °C. The PON1 enzyme activities (EU) were calculated according to the following formula^36^.

$$\begin{array}{c} \text{EU }=\;\left[\text{reaction volume }\left(\text{mL}\right)\;\times \text{ dA}/\text{dT }\times \text{ 1},\;000\right]/\\ {}[\varepsilon \;\times \text{ enzyme volume }\left(\text{mL}\right)\;\times \text{ d }\left(\text{cm}\right) \end{array}$$

## Results and discussion

In this study, our main objective is to investigate the effects of PON1 activity on the proliferation and aggressiveness of glioblastoma cancer by measuring the PON1 activity of glioma cells of the U87MG cell line treated with TQ, AZA, and EF-24. We also know that comprehensive and in-depth research will help to understand the closest mechanisms of PON1 in cancer, cardiovascular diseases, neurological diseases and inflammatory diseases. Our current research is focused on evaluating the effects of PON1 enzyme activity and glioma cell behaviour with TQ, AZA, and EF 24.

On the basis of antioxidant mechanisms, the enzymes superoxide dismutase (SOD), catalase (CAT), glutathione peroxidase (GPx) and glutathione reductase (GRx) play a crucial role in scavenging free radicals^37^. Among these enzymes, PON1 is also a candidate antioxidant enzyme whose effective antioxidant properties are associated with high-density lipoprotein (HDL)^38^.

Since PON1 inactivates oxidised LDL phospholipids, it also prevents the oxidation of phospholipids to HDL^39^. In the context of this ability, PON1 could, therefore, be a potential mediator of fat-soluble radicals^40^. Maintaining a powerful bridge between antioxidants and oxidants^41^ is therefore generally considered to have a strong link to the prevention of cancer development^42^.

Xenobiotics can reach the brain, but the causes of brain tumours are still not well understood and influenced by them. However, we know that the brain is able to metabolise xenobiotics because it has a cytochromes P450 (CYPs) monooxygenase system. The metabolism of xenobiotics is renamed in other terms to reduce the reactive and toxic metabolites in neuronal cells^17^.

Depending on the location of PON1, the liver, and the blood have a suitable foundation to prevent ROS species from being synthesised by PON1. Since the hydrolysis of acetylcholinesterase-inhibiting oxides has a better chance of detoxifying the poisoning effects of xenobiotics with higher paraoxonase1 activity, PON1 acts so strongly through the reactive oxygen species.

**Figure 1.**
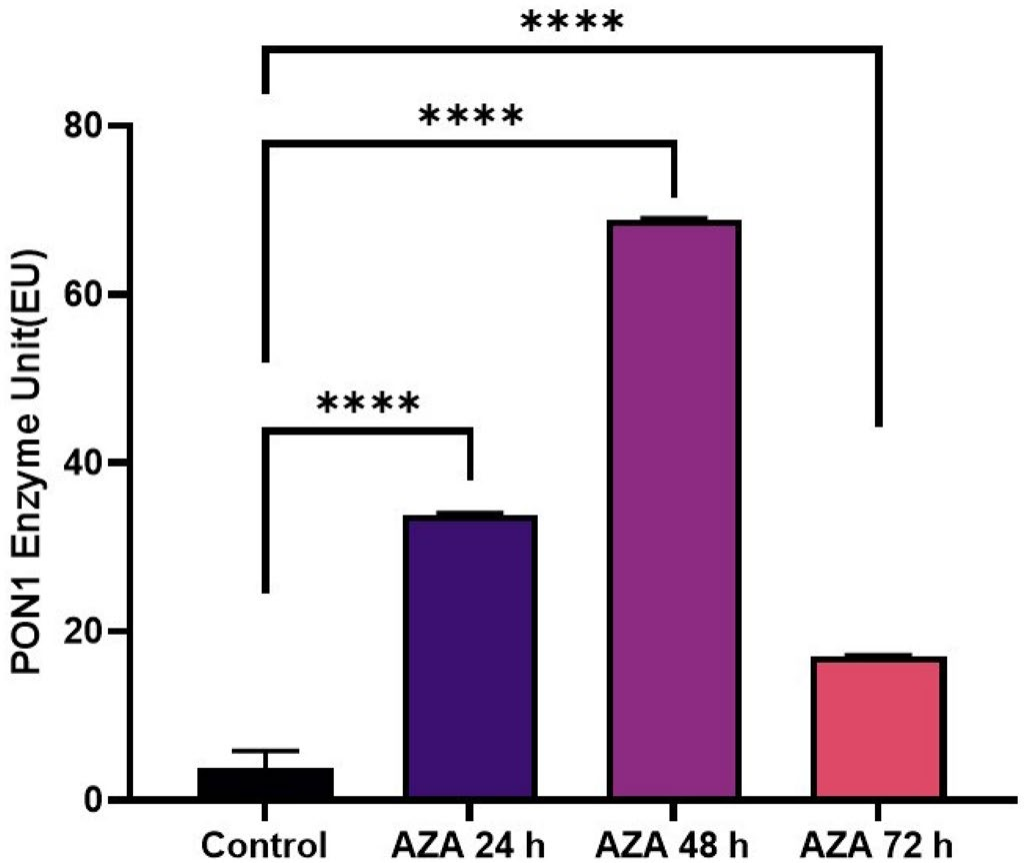
Modulating effects of AZA on PON 1 enzyme activity in human glioblastoma cells U87MG.

**Figure 2.**
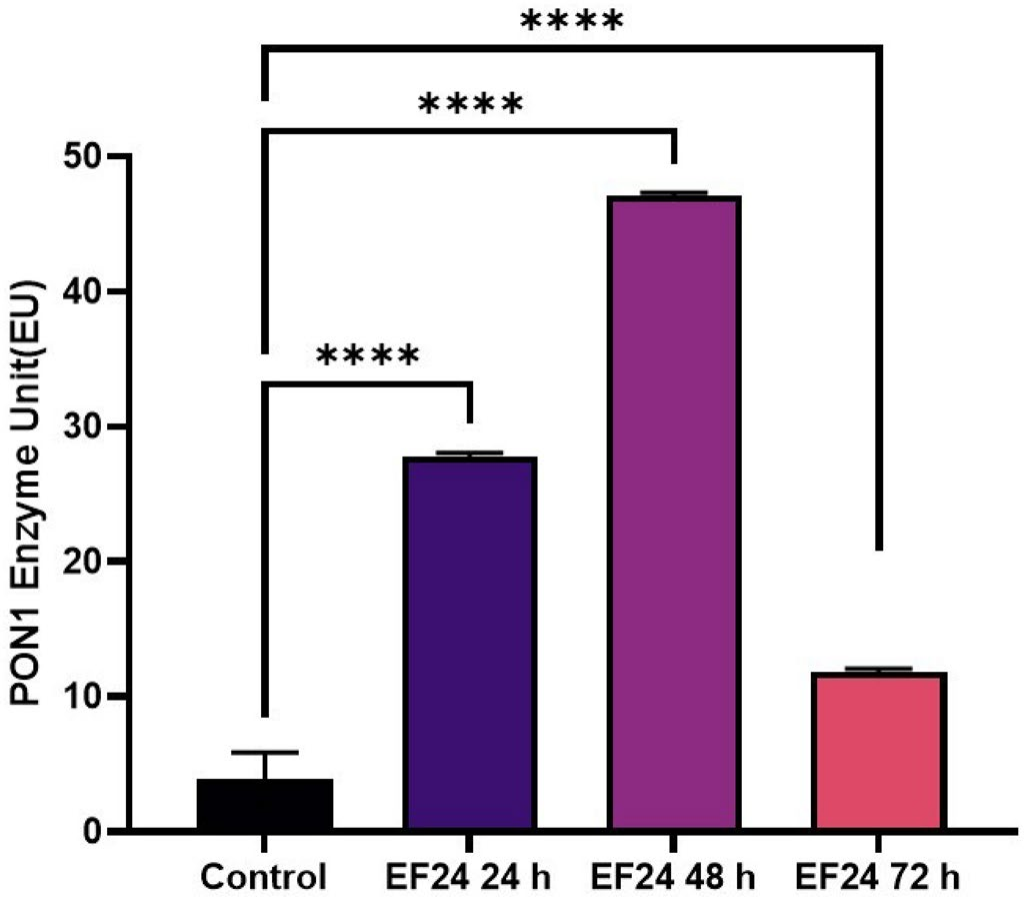
Modulating effects of EF 24 on PON 1 enzyme activity in human glioblastoma cells U87MG.

**Figure 3.**
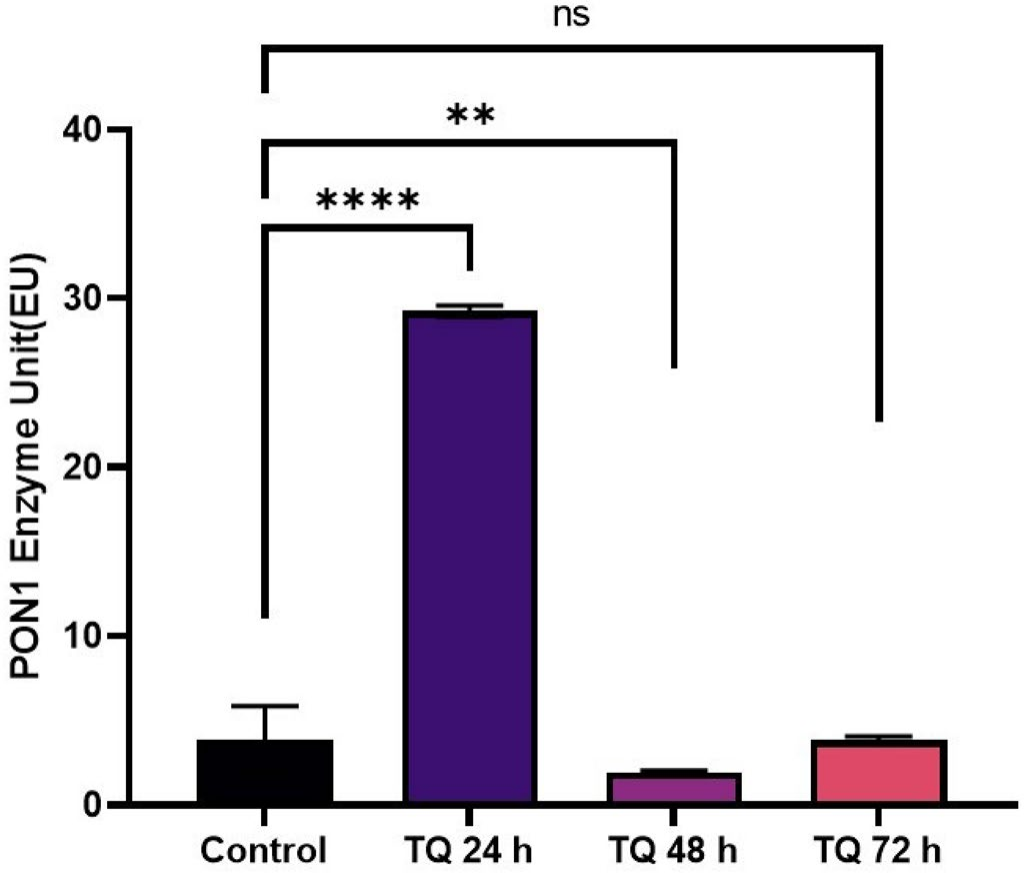
Modulating effects of TQ on PON 1 enzyme activity in human glioblastoma cells U87MG.

Je-Yoel Cho conducted a study to determine PON1 protein status and reduced PON1 enzyme levels during proteolytic degradation in blood. In that study, it was postulated that lung cancer cells could take up PON1 from the blood because of its antioxidant activities to prevent oxidative stress with reduced PON1 serum levels^43^. Therefore, in this preliminary study, we tested the PON1 activity of cells treated with TQ, AZA, and EF-24 from lysates of the glioblastoma cell line U87MG (Figure1-3). In this context, the concentrations of TQ, EF-24, and AZA were determined to evaluate the metabolic activity of the cells using the MTT assay. According to the MTT assay, when treated with the appropriate dosage, the results showed increased PON1 activity when treated with TQ and EF-24 in the U87MG cell line (*p* < 0.0001). According to the results, the IC50 value of TQ application in the U87 MG cell line was set at 67.03 µM at the 72^nd^ hour. (*p* < 0.05). The IC50 value of TQ application in the U87 MG cell line was found to be 67.03 µM at the 72^nd^ hour (*p* < 0.05). We also used AZA to compare the effects of TQ and EF24 on the PON1 enzyme. In our current study, the IC50 value of AZA application could not be observed in the U87 MG cell line at the 72^nd^ hour in this study, but AZA was found at 100 µM in the U87 MG cell line in the literature^44^. In one study, the Li group found that AZA can suppress tumour metastasis and angiogenesis in vivo^45^^,^ but the mechanism of this finding is not yet clear. Treatment with AZA could be a candidate for cancer therapy. Also, the Li group postulates that AZA can delay tumour progression in vivo.

Another study was conducted by Ray’s group, which treated human glioblastoma cells T98G and U87MG with AZA. Their results strongly suggest that treatment with AZA cannot stop the efficacy of temozolomide (TMZ), but that AZA also acts as a modulator in human glioblastoma cells^46^.

According to our findings in this study with decreased PON1 activity, glioma cancer cells might have PON1 from the blood to utilise its antioxidant activities that maintain a balance to prevent oxidative stress during cancer progression. An increase in PON1 enzyme activity was observed after treatment with TQ and EF-24 (Figure 1–3). The high PON1 enzyme activity suggests that TQ and EF-24 support PON1 in its crucial role as an antioxidant enzyme in cancer development. Therefore, TQ and EF-24 can be used as medicinal herbs to reduce the effects of ROS species by binding PON1. However, a study by Demir et al. confirms low PON1 enzyme levels in patients with malignant gliomas^47^. ROS can act as a defect in the antioxidant system, which can lead to cancer growth at high levels. Tseng et al. also found that PON2 was highly expressed in glioblastoma multiforme (GBM) cells compared to normal brain tissue. In their study, they also investigated whether the overexpression of PON2 leads to a reduction in the ROS concentration. Interestingly, they found that the ROS concentration in GBM cells overexpressed with PON2 was significantly reduced compared to controls^48^.

The incredible power of PON1 activity as an antioxidant is present under various conditions such as diet and lifestyle habits as well as environmental toxins. The effect of PON1 in preventing reactive oxygen species has been widely recognised in carcinogenesis. The ability of PON1 definitely maintains the antioxidant and anti-inflammatory mechanisms in the brain. Consequently, PON1 also has a facilitated pathway into the brain to assist the CYPs system in detoxifying toxins and plays a role in carcinogenesis. However, the maintenance of reduced enzyme activity requires some active ingredients, such as medicinal herbs like TQ and EF 24, to demonstrate the prominent role of PON1.

## Conclusions

However, these possibilities created by this preliminary study need to be confirmed by further experiments with other herbs that may reduce cancer risk. Thyme contains terpenoids, a group of phytochemicals that act as antioxidants and play a crucial role in protecting against cancer. Curcumin analogs with a five-carbon bond between the two phenyl rings, known as diarypentonoids, also have the same effect against free radicals. It is postulated that abnormal fucosylation is associated with all aspects of cancer biology. Another pathway of fucosylation may represent a new strategy for cancer treatment with some PON1-related herbs. In further studies, we also want to investigate some other antioxidant enzyme activities related to CYPs enzymes in glioma cells with some herbs.

## Data Availability

The authors confirm that the data supporting the results of this study are available in the article. Raw data supporting the results of this study are available upon reasonable request to the corresponding author
